# Editorial: Immune aging and its consequences

**DOI:** 10.3389/fendo.2023.1250141

**Published:** 2023-07-28

**Authors:** João Moura

**Affiliations:** Centre for Neuroscience and Cell Biology, University of Coimbra, Coimbra, Portugal

**Keywords:** aging, inflammaging, chronic inflammation, inflammatory cytokines, immune dysregulation

Immune aging is a compromise between the age-associated immune damage induced by cumulative immune stimulation and an attempt to maintain immune tolerance throughout the individual’s adult life. It is mainly a consequence of the increased human life expectancy, and its incidence thus directly correlates with higher life expectancy (1).

Immune aging is associated with a decline in immune response potency, as was recently demonstrated during the COVID-19 pandemic (2) and with the increased incidence of autoimmune diseases (3), due to the accumulation of a diverse set of effector T-cell populations that release excessive amounts of pro-inflammatory cytokines (4). This phenomenon, called inflammaging, is associated with various chronic inflammatory conditions affecting all tissues throughout the human body, from cerebrovascular inflammation (5) to diabetic foot ulcerations (6).

Attempts to slow down or even reverse specific age-induced immune changes look promising due to the multiple connections with all the other systems in the human body (7), but are still far from clinical use.

In this Research Topic dedicated to immune aging and its consequences, we cover some of the molecular mechanisms associated with inflammaging and some of its consequences, in particular in the context of diabetes.

Regarding the possible causes of inflammaging, Liu et al. report on the identification of Vsig4 (V-set and immunoglobulin domain containing 4) as the critical checkpoint gene in offsetting age-associated hypertension and diabetes. This data originated from a Chromogranin A (CgA) knockout mouse model for healthy aging that does not loose insulin sensitivity and develop hypertension with age, leading the authors to conclude that inflammaging may also derive from the accumulation of bacterial DNA-induced inflammation. Additionally, Huang et al. review the mechanisms of macrophage dysregulation in diabetic skin, proposing an early screening and evaluation tool for diabetic foot based on skin macrophage numbers and functional attributes, specifically pro-inflammatory cytokine production.

Additionally, Du et al. have reviewed the multifunctional role of osteopontin as a proinflammatory immunochemokine widely involved in the aging processes of multiple organs and tissues; it is of importance in various pathological processes associated with immune aging, such as atherosclerosis, osteoporosis, neurodegenerative disorders, and retinal aging. The authors further discuss the molecular mechanisms of osteopontin action in different tissues and cells, supporting the development of osteopontin-targeted therapeutic strategies to fight the spread of age-related diseases.

Regarding the consequences of immune aging, Li et al. explore the impact of diabetes and cognitive impairment in the older Chinese population. In this study, which involved a large cohort of 4499 individuals, Li et al. show that cognitive impairment and diabetes (both associated with inflammaging) inversely correlate with survival in this cohort, synergistically more than tripling the mortality for individuals above 60 years old.

On the other hand, Wang et al. present very interesting data that shows that the metabolite itaconate, produced by macrophages in the context of inflammaging, inhibits osteoclast differentiation and activation in an induced inflammatory bone loss animal model. If this mechanism translates to humans, itaconate may be a key target in osteoporosis by regulating osteoclast-mediated bone homeostasis.

In conclusion, addressing immune aging has never been so urgent due to the continuous change in demographics towards a more generalized increase in human life expectancy and the implications of the chronic low-grade inflammation associated with immune aging for various pathologies that preferentially affect the expanding elderly population.

## Author contributions

The author confirms being the sole contributor of this work and has approved it for publication.
